# Exploring the relationship between environmental enteric dysfunction and oral vaccine responses

**DOI:** 10.2217/fmb-2018-0016

**Published:** 2018-06-21

**Authors:** James A Church, Edward PK Parker, Margaret N Kosek, Gagandeep Kang, Nicholas C Grassly, Paul Kelly, Andrew J Prendergast

**Affiliations:** 1 Zvitambo Institute for Maternal & Child Health Research, Harare, Zimbabwe; 2 Centre for Genomics & Child Health, Blizard Institute, Queen Mary University of London, UK; 3 Department of Infectious Disease Epidemiology, St Mary's Campus, Imperial College London, London, UK; 4 Department of International Health, Johns Hopkins Bloomberg School of Public Health, Baltimore, MD 21205, USA; 5 Department of Gastrointestinal Sciences, Christian Medical College, Vellore, Tamil Nadu, India; 6 Tropical Gastroenterology & Nutrition group, University of Zambia School of Medicine, Lusaka, Zambia

**Keywords:** EED, infant, oral vaccine

## Abstract

Oral vaccines significantly underperform in low-income countries. One possible contributory factor is environmental enteric dysfunction (EED), a subclinical disorder of small intestinal structure and function among children living in poverty. Here, we review studies describing oral vaccine responses and EED. We identified eight studies evaluating EED and oral vaccine responses. There was substantial heterogeneity in study design and few consistent trends emerged. Four studies reported a negative association between EED and oral vaccine responses; two showed no significant association; and two described a positive correlation. Current evidence is therefore insufficient to determine whether EED contributes to oral vaccine underperformance. We identify roadblocks in the field and future research needs, including carefully designed studies those can investigate this hypothesis further.

Although the last decade has witnessed impressive declines in the global burden of diarrheal disease [1], it remains the second largest infectious cause of child mortality with 578,000 child deaths annually [2]. Furthermore, diarrhea causes extensive morbidity, particularly in young children, with an estimated 957.5 million episodes of disease and 7506 thousand years lost due to ill health in 2016 [3].

A major contributor to the global reduction in diarrhea has been improved coverage of oral rotavirus vaccination, which had been introduced in 93 countries by November 2017 [4]. Oral vaccination is also a key prevention strategy against enteric bacterial pathogens such as *Vibrio cholerae* and *Salmonella typhi*; and vaccines against other bacteria including *Shigella* and *Campylobacter* are in development. Meanwhile the sustained role of oral poliovirus vaccine (OPV) in the polio endgame strategic plan further emphasizes the potential impact of oral vaccines in mitigating enteric infections. Oral vaccines obviate the need for needles, are easy to administer and generate a local immune response in the gut, thereby conferring protection and reducing the likelihood of onward transmission. However, the success of two of the available oral vaccines (rotavirus and polio) also hides a troublesome truth: they significantly underperform in low-income compared with high- and middle-income countries [5].

Stanley Plotkin noted as early as 1960 that Congolese infants were less likely to develop neutralizing antibodies to an attenuated type 1 poliovirus vaccine compared with counterparts in Europe and the USA [6]. Seroconversion to OPV has remained consistently lower in low-income compared with developed countries [7], which has hampered global eradication efforts. Poor immunogenicity has also been reported for both live and killed oral cholera vaccines [8]. Finally, oral vaccine efficacy against severe rotavirus gastroenteritis is substantially lower in sub-Saharan Africa (39.3%, 95% CI: 19.1–54.7) [9] and Asia (48.3%, 95%CI: 22.3–66.1) [10] than in Europe/USA (85–98%) [11,12].

Although a number of factors have been proposed to explain this efficacy gap, the precise biological mechanisms have not been fully elucidated [13]. Notably, parenteral vaccines do not display diminished efficacy in these same contexts suggesting that systemic immunodeficiencies are not underlying these attenuated responses. As a result, factors associated with the intestinal milieu have been proposed as critical determinants. These include interference from maternally acquired antibodies [14], early infection with enteropathogens [15], glycans expressed on the gut epithelium [16], micronutrient and macronutrient deficiencies [17], intestinal dysbiosis [18], aflatoxin exposure [19] and a subclinical disorder of small intestinal structure and function termed environmental enteric dysfunction (EED) [20]. In this review, we critically evaluate the evidence supporting the role of EED in reducing oral vaccine immunogenicity (Box 1) and highlight the current research gaps and future opportunities.

## Environmental enteric dysfunction

EED is a subclinical condition affecting the small bowel. It is almost ubiquitous among children living in poverty, among whom oral vaccines have consistently underperformed [20]. It is characterized morphologically by the presence of intestinal villous flattening, crypt hyperplasia and a chronic T-cell-mediated inflammatory enteropathy [21,22]. Structural changes are accompanied by functional disturbances of increased intestinal permeability and reduced absorptive capacity [23–25]. EED was first reported many decades ago as an incidental finding on small intestinal biopsies (then termed ‘tropical enteropathy’) [26], but has garnered renewed interest in recent years, with researchers seeking to better define its pathogenesis and consequences and to evaluate preventive or curative interventions.

The etiology of EED remains elusive. Intestinal biopsies taken from stillborn fetuses indicate that EED is not present at birth [27] but rather develops early in infancy and appears to persist into adulthood. Studies of expatriates and migrants have shown that EED can be reversed by a change in environment, specifically by transfer from an environment of poor water and hygiene to the USA or Europe [28,29]. Moreover, there is a wealth of observational data supporting a role for microbial exposure in the development and persistence of EED. Children living in conditions of poor hygiene with sustained exposure to feco–oral contamination are more likely to develop EED than children living in less contaminated households [30]. A recent mouse model of microbial exposure and moderate malnutrition, which recapitulates the steps needed to invoke EED, supports this [31]. Children in low-income countries also experience a high multiplicity of infections even with no diarrhea [15]. However, while specific organisms have been associated with deranged gut biomarkers [32–35], no single pathogen has been implicated to date as singularly important. Instead, it is likely that persistent exposure to multiple enteropathogens is important. Small intestinal bacterial overgrowth (SIBO), which has been correlated with biomarkers of EED in Bangladeshi children [36], occurs in a proportion of children living in impoverished settings, and its relationship to EED remains to be elucidated [37]. In addition to carriage of pathogenic organisms, changes in the commensal microbiota may also play a role. A recent study of 81 Malawian children showed that the fecal microbiota differs across a spectrum of EED severity (specifically three categories of gut permeability defined by lactulose:mannitol ratios [38]). The three genera *Megasphaera, Mitsuokella* and *Sutterella* were more abundant in severe EED versus no EED while the genera *Succinivibrio, Klebsiella* and *Clostridium XI* were less abundant.

A number of obstacles limit our understanding of EED. Consensus on a formal case definition for EED is currently lacking. There are ethical and practical constraints to obtaining small intestinal biopsies from asymptomatic young children; studies therefore tend to rely on a limited range of noninvasive biomarkers to identify alterations in gut structure and function. It has been argued that biomarkers capable of detecting early changes in intestinal inflammation or enterocyte mass could ultimately become more sensitive than direct visualization to measure the condition and how it evolves [39]. Indeed, other chronic intestinal disorders, such as inflammatory bowel disease, employ biomarkers, although these are generally used in conjunction with endoscopic surveillance. In contrast, because biopsies are difficult to obtain in EED, very few corroborative studies have compared noninvasive biomarkers with histological changes; where it has been done in adult populations, they do not appear to correlate well [40,41].

We do not yet fully understand the exposures that underpin EED, the timing of onset and whether the changes in the small bowel that characterize EED are deleterious or adaptive for young children who are repeatedly exposed to multiple enteric pathogens. We use the term EED throughout this paper although we recognize an ambiguity about whether the condition comprises a ‘dysfunction’. Rather than being a distinct condition with a single etiology, EED may be the final common pathway arising from multiple overlapping and interacting insults [20]. A single biomarker will probably never capture this. Similarly, it is unlikely to be biologically relevant to categorize the binary presence or absence of EED; rather, there appears to be a population-level alteration in gut structure and function of severity, which varies over time and appears to be especially important in young children.

## EED & oral vaccine failure

Several findings have led to speculation that EED may shape geographic trends in oral vaccine underperformance [42,43]. First, EED evolves during the period when oral vaccines are administered in early infancy. Second, oral vaccines are more likely to fail in low-income countries [44], where EED is prevalent. Third, oral vaccine failure is prone to seasonal variations [45] in a manner that has also been described for EED [40]. A plausible rationale for EED as a cause of oral vaccine failure also exists at the biological level. The intestinal mucosa is hugely dynamic, with important functions as a mechanical, antimicrobial and immunological interface. Widespread and profound alterations in gut structure and function may interfere with the processing of an oral vaccine as it transits through the small intestine.

**Figure 1.**
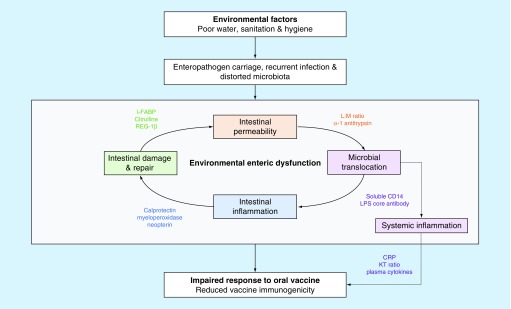
**Proposed biological pathway demonstrating the hypothesized role of EED (incorporating four domains depicted by four colours) in the underperformance of oral vaccines.**

At present, there is broad consensus that a range of EED biomarkers will provide the most informative measure of associations between gut dysfunction and oral vaccine response [46–48]. Four pathological domains have been proposed (Table 1) [39]: intestinal permeability; intestinal epithelial damage and repair; intestinal inflammation; and microbial translocation and immune activation. Incorporating each of these domains into a conceptual framework, we outline mechanisms through which disturbances in these domains might influence oral vaccine ‘take’ (Figure 1). Intestinal epithelial damage, for example, may alter Peyer's patches and the processing efficiency of the mucosal immune interface. Intestinal inflammation may be characterized by a proinflammatory mucosal response together with increased numbers of immunomodulatory molecules acting as inhibitors, increased numbers of Tregs and enhanced dendritic cell function [49,50]. However, the degree to which each domain contributes to the pathogenesis of oral vaccine failure likely varies. In the following section, we evaluate each of these domains of EED in turn and the evidence for their contribution to oral vaccine failure in low-income countries.

**Table 1.** **Four domains of biomarkers used to measure environmental enteric dysfunction.**

**Domains**	**Biomarkers**
Intestinal permeability	D-xylose, mannitol or rhamnose absorption, lactulose paracellular uptake, lactulose:mannitol ratio, AAT leakage into gut lumen, zonulin

Intestinal epithelial damage & repair	I-FABP, plasma citrulline and/or conversion of alanyl-glutamine to citrulline, lactose tolerance test (as marker of brush border damage), fecal lipocalin, *Fecal Reg1B* (epithelial cell renewal)

Intestinal inflammation	Stool calprotectin, MPO, lactoferrin, neopterin

Microbial translocation & systemic immune activation	Plasma LPS core antibody and/or LPS binding protein, circulating soluble CD14, KT ratio, plasma cytokines, CRP

Other	Hydrogen breath testing (as a measure of SIBO)

Biomarkers not detailed in Keusch review table are labeled in italics.

AAT: α1-anti-trypsin; CRP: C-reactive protein; I-FABP: Intestinal fatty acid binding protein; KT ratio: Kynurenine–tryptophan ratio; LPS: Lipopolysaccharide; MPO: Myeloperoxidase; SIBO: Small intestinal bacterial overgrowth.

Adapted from [39].

### Search method

We conducted a systematic search using Medline (see Appendix 1) for articles published on or before 2 May 2017 describing vaccine responses in the context of EED. Abstracts and titles from all years were compiled in Endnote (Thomson Reuters) and screened. We extracted data on study year, country, participant number and age, vaccine type, choices of EED biomarkers, measurement time points and analysis. The search was subsequently broadened also to include studies detailing oral vaccine responses in the context of other gastrointestinal conditions with phenotypic features in common with EED. Our review was restricted to articles either written in English or translated into English. Unpublished data were not included; however, two studies published during the course of the review were subsequently included.

## Results overview

We identified eight studies describing oral vaccine responses in the context of EED across six countries and three continents (South America, Africa and India). Six studies describe immune responses to OPV and/or rotavirus vaccine (RVV) in infants (under 1 year of age) and two studies focus on responses to oral cholera vaccine in older children aged 3–14 years (Table 2).

**Table 2.** **Summary of studies evaluating association between environmental enteric dysfunction and oral vaccine responses.**

**Author**	**Country**	**N**	**Age at enrollment**	**Vaccine (schedule)**	**Outcome measure (definition)**	**Timing of measure**	**Age of EED measure (timing)**	**Power**	**Domains**	**Biomarkers measured**	**Definition of EED**	**Authors’ interpretations**	**Ref.**
Becker-Dreps	Nicaragua	43	3 months	RV5 (2 months)	Seroconversion (RV IgA ≥fourfold rise)	1 month post first dose vaccine	2 months (day of first dose vaccine)	NS	2	Stool AAT, calprotectin, MPO, Neopterin	Kosek score; ‘4 Biomarker EE score’	Negative association	[51]

Bucardo	Nicaragua	92	2 months	RV1 or RV5 (2 months)	Seroconversion (RV IgA ≥fourfold rise)	1 month post first dose vaccine	2 months (day of first dose vaccine)	NS	1	Stool calprotectin	NS	No association	[16]

Grassly	India	291	6–11 months	mOPV-3 (D14)	Seroconversion (serotype 3 poliovirus-specific NT ≥1 in 8)	21 days post vaccine	6–11 months (day of vaccine)	Subset from trial	4	Stool AAT, calprotectin, MPO, neopterin; plasma I-FABP, EndoCAB, sCD14	Kosek score; binary score ( = 1 if 1 or more biomarker measurements in top quartile, = 0 if otherwise)	No association	[52]

					Shedding (fecal poliovirus detection	7 days post vaccine							

Kosek	Peru	173	7 months	tOPV (2, 4, 6 months)	OPV failure (log2NT <3)	1 month post last dose vaccine	3 months (1 month post first dose vaccine)	NS	2	Plasma citrulline, KT ratio	NS. Testing candidate biomarkers	Negative association	[53]

Lagos	Chile	178	5–9 years	*Orochol* (CVD 103) (D1)	Seroconversion (vibriocidal AB titre rise ≥4)	1 day pre, 10 days post vaccine	5–9 years (1 day pre vaccine)	Yes	1	H2 breath test (proximal SI)	NS	Negative association	[54]

Mwape	Zambia	142	6–12 weeks	RV1 (6,10 weeks)	Seroconversion (RV IgA ≥fourfold rise)	1 month post last dose vaccine	6–12 weeks (day of first dose vaccine)	Yes	3	Plasma zonulin, I-FABP, EndoCAB, sCD14	NS	Positive association	[55]

Naylor	Bangladesh	261	0–7 days	RV1 (10, 17 weeks)	Rotarix protection	Absence RV diarrhea between 18 & 52 weeks	6, 12 or 18 weeks (2 weeks post first dose)	NS	4	Urine LM; stool AAT, calprotectin, MPO, neopterin, REG1B; plasma EndoCAB, sCD14, CRP	NS. Used clusters of biomarkers	Negative association	[56]

					Seroconversion (RV IgA >20 U/ml)	1 week or 35 weeks post last dose vaccine							

		509	0–7 days	tOPV (6,10,14 weeks)	Seroconversion (NT ≥threefold rise)	4 weeks post last dose vaccine							

Uddin	Bangladesh	40	3–14 years	Dukoral (WC-rBS); (D0,14)	Vibriocidal, LPS & CTB-IgA, IgM & IgG	21 days post first dose vaccine	3–14 years (day of first dose vaccine)	NS	4	Stool AAT, MPO; plasma I-FABP, EndoCAB, sCD14	NS	Positive association	[57]

AAT: α1-anti-trypsin; CRP: C-reactive protein; CTB: Cholera toxin subunit B; D1: Day 1 of study; EE: Environmental enteropathy; EED: Environmental enteric dysfunction; I-FABP: Intestinal fatty acid binding protein; KT ratio: Kynurenine–tryptophan ratio; LM: lactulose-mannitol; LPS: Lipopolysaccharide; mOPV-3: Serotype-3 monovalent; MPO: Myeloperoxidase; NS: Not stated; NT: Neutralizing titre; OPV: Oral poliovirus vaccine; RV: rotavirus; RV1: Monovalent rotavirus vaccine or Rotarix; RV5: Pentavalent rotavirus vaccine or RotaTeq; SI: Small intestine; tOPV: Tetravalent OPV.

**Figure 2.**
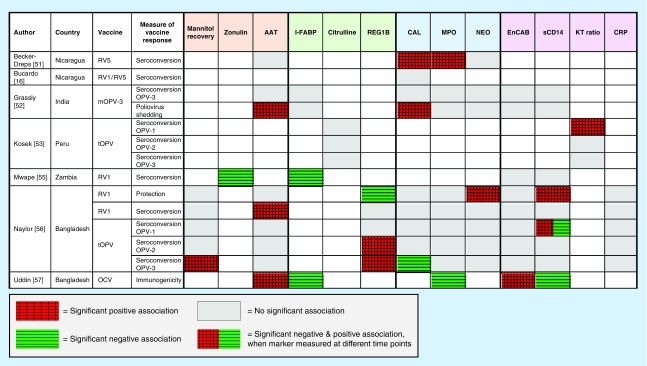
**Summary of evidence for association between markers in four domains of environmental enteric dysfunction and oral vaccine response.**

These studies used a range of biomarkers to characterize EED, with each selecting a different combination of markers measured at various time points (Table 2). Three studies included at least one biomarker from all four domains. Four of the eight studies reported overall a negative association between EED and oral vaccine responses, with elevated markers of EED associated with reduced oral vaccine performance [51,53,54,56]. Two studies showed no significant association [16,52] and two studies concluded that EED was positively correlated with immunogenicity to oral vaccine antigens [55,57]. The majority of studies relied on vaccine immunogenicity as a proxy for oral vaccine efficacy. The results of these studies are summarized in Figure 2 (with additional data detailing ranges and effect sizes available in Appendix 2).

### Domain 1 – intestinal permeability

Dual-sugar tests have been regarded as the gold standard for assessing intestinal permeability. They rely on measuring the fractional excretion of nonmetabolizable saccharides of varying sizes (most commonly lactulose and or mannitol) in urine after oral ingestion. The smaller sugar, mannitol, should be absorbed by healthy villi and be detected in the urine, while the larger sugar, lactulose, should not be absorbed due to intact tight junctions between enterocytes. Urinary lactulose recovery, or an elevated lactulose:mannitol ratio, may therefore reflect increased intestinal permeability. However, the test has several limitations [58]: first, it is time-consuming, requiring a period of fasting and prolonged urine collection, which is difficult in young infants; second, the ingested solute load may alter intestinal transit time and permeability; and third, variations in test procedure and analysis make cross-study comparisons challenging. Only one study reported a dual-sugar test in relation to oral vaccine responses (Figure 2). In a cohort of 509 Bangladeshi infants, urinary mannitol levels measured at 3 months of age were negatively associated with OPV3 seroconversion [56]; however, no association was found for other strains of OPV, neither with Rotarix immunogenicity nor protection from rotavirus diarrhea.

α-1 antitrypsin (AAT) is a plasma protein synthesized by the liver. It is not normally detectable in the stool of healthy subjects and its presence indicates leakage of AAT from the blood to the intestinal tract due to increased gut permeability. However, quantifying the degree of intestinal permeability is problematic because plasma levels of AAT are prone to variation between subjects as it is an acute phase reactant. In the context of vaccine responses, AAT concentration has been the most frequently used marker of gut permeability. Four studies of three different oral vaccines have examined associations between oral vaccine responses and AAT concentrations (Figure 2). Two large studies (N >250) in Bangladesh and India found no association between seroconversion to OPV and AAT (measured at 3 months of age in Bangladesh and between 6–11 months of age in India). The Bangladesh study reported a negative association between RV1 seroconversion and AAT; however, AAT concentrations did not correlate with Rotarix protection from rotavirus diarrhea (recorded up to the age of 1 year in vaccinated infants). In another study of RotaTeq in Nicaragua there was no association between seroconversion and AAT, although the sample size in this study was small (N = 43) [51]. Finally, in another cohort of older Bangladeshi children, stool AAT was negatively associated with IL-10 (regulatory) T-cell responses specific to cholera toxin (p = 0.02) [57]; however, there was no significant association with more widely used measures of cholera vaccine response (vibriocidal antibodies or lipopolysaccharide [LPS] and cholera toxin subunit B [CTB] antibodies).

Zonulin is a protein that binds to specific receptors on intestinal epithelial cells inducing the disassembly of tight junctions and thereby increasing permeability. One study, among Zambian infants, measured zonulin levels at the time of oral vaccine administration and showed a positive association between zonulin and subsequent Rotarix seroconversion [55]. Therefore, in contrast to the other four studies showing no or negative associations, this study is the first to propose that increased intestinal permeability may actually enhance oral vaccine seroconversion.

### Domain 2 – enterocyte mass & function

Several biomarkers reflect damage, repair and mass of the intestinal epithelium. Intestinal fatty acid binding protein (I-FABP), found in mature enterocytes of the small and large intestine, is released into the serum when the cell membrane is compromised [56]. Owing to its short half-life, it is a dynamic measure of acute enterocyte damage but may be less informative of chronic injury. Two studies found a positive association between levels of I-FABP and oral vaccine responses. One with cholera toxin-specific effector memory T cells in Bangladeshi children, although this association was not observed for other immunological markers of cholera response [57]; the other, with rotavirus IgA in Zambian infants [55]. Together, they suggest that gut epithelial injury can enhance immune responses to oral cholera and rotavirus vaccines, respectively. However, another study measuring I-FABP in Indian children 6–11 months of age, reported no associations with OPV3 seroconversion or shedding (Figure 2) [52].

Regenerating gene 1B (Reg-1B) is a protein measurable in stool, which has been implicated in the regeneration and repair of the intestinal epithelium [56]; and is upregulated in response to enteric infections [59] and inflammatory conditions [60]. Only one study, conducted in Bangladesh, reports Reg-1B in relation to oral vaccine response [56]. Associations with Reg-1B were found in opposite directions for OPV and Rotarix: elevated Reg-1B at 6 and 12 weeks of age was associated with failure to seroconvert to OPV2 and OPV3, respectively, but with protection from rotavirus diarrhea. Despite this, the authors conclude overall that EED is associated with oral vaccine underperformance.

Citrulline is produced mainly by enterocytes in the small bowel and provides a measure of enterocyte mass [61], independent of nutritional status and inflammation [61]. We identified only one study, which found no associations between citrulline and OPV response in 173 Peruvian children [53]. No studies have evaluated plasma zonulin (a biomarker of tight junction function) or fecal lipocalin (a biomarker of intestinal epithelial damage) in the context of oral vaccine performance.

### Domain 3 – intestinal inflammation

Biochemical measures of intestinal inflammation have been widely used in clinical practice and research as diagnostic and surveillance tools for enteric infections and inflammatory bowel disease [62]. Fecal calprotectin and myeloperoxidase (MPO) are both products of neutrophils, and neopterin is a product of macrophages and dendritic cells. The benefit of these biomarkers is that they are robust and easily reproducible with commercially available assays; however, to date there are no reference ranges for these values among children in low-income settings.

Five studies describe levels of gut inflammatory biomarkers in relation to oral vaccine responses (Figure 2). Among 590 Bangladeshi infants aged 3 months in the PROVIDE study, low levels of fecal neopterin were associated with Rotarix success (no rotavirus diarrhea in the year following vaccination) suggesting a detrimental effect of a proinflammatory milieu on vaccine performance or that children who avoided a recent enteric virus infection had a better vaccine take. In the same study, increased calprotectin was associated with successful OPV3 seroconversion [56], which could be consistent with an enhancing effect of intestinal inflammation. However, other studies do not support this and even describe the converse. One study in Nicaragua showed no association between calprotectin and seroconversion to either Rotarix or RotaTeq [16]. In a trial of azithromycin in India, fecal calprotectin was significantly lower among infants who shed poliovirus 7 days after OPV administration (although an association was not found for seroconversion) [52]. Finally, in a different study of Nicaraguan infants, elevated calprotectin at 2 months of age was associated with reduced seroconversion to RotaTeq [57]. Among the same Nicaraguan infants, elevated MPO was also associated with failure to seroconvert to RotaTeq*.* However, there was no significant association between MPO and OPV3 response in the India trial or with OPV and rotavirus responses in the PROVIDE study [52,56]. Intriguingly, among older children in Bangladesh, elevated MPO was associated with improved responses to an oral cholera vaccine [57].

An alternative strategy when examining associations with EED involves grouping together biomarkers as in prior studies of stunting. The Mal-ED study showed that a combination of three fecal markers outperformed any single marker in predicting linear growth deficits [63]. Similarly, in a cohort of 6–26 month-old children in north-east Brazil, the combination of MPO and neopterin added power in predicting subsequent growth impairment compared with MPO alone [64]. The clustering of biomarkers into an EED ‘activity score’ has also been used to explore associations with vaccine responses. In one study, the median EED ‘activity score’ was higher in Nicaraguan infants who did not seroconvert to RotaTeq compared with those who did (5.0 vs 3.5, respectively; p = 0.03) [51]. By contrast, there was no association between OPV3 seroconversion and this disease activity score in 293 Indian infants enrolled in the azithromycin trial [52]. It is worth noting that levels of intestinal inflammation measured in the Nicaraguan infants [51] were much lower on average compared with the studies from the Indian subcontinent. For example, among the 590 Bangladeshi infants in the PROVIDE study, levels of fecal calprotectin and MPO at 3 months of age were elevated in 82.7 and 88.1%, respectively (using normal values based on Western standards) [56], highlighting the high prevalence of intestinal inflammation among young children in low-income countries.

Taken together, the evidence for intestinal inflammation altering oral vaccine responses is heterogeneous among the few available studies. The positive associations described in two studies between intestinal inflammation and vaccine response raise the possibility that an inflammatory intestinal milieu, perhaps counterintuitively, favors a robust immune response; however, this requires further evaluation.

### Domain 4 – microbial translocation & immune activation

There is a lack of reliable assays to detect microbial translocation. LPS (or endotoxin) is a key constituent in the membrane of Gram-negative bacteria, and therefore a plausible measure of translocation from the gut; however, it is difficult in young infants to avoid endotoxin contamination during venepuncture. Alternatively, IgM or IgG specific to the core domain of endotoxin (EndoCAb) is an indirect measure of LPS exposure; however, there have been technical problems with the commercially available assay [65]. Soluble CD14 (sCD14), which acts as a co-receptor in the LPS/TLR4 signal transduction pathway, is a marker of monocyte activation, particularly in response to endotoxin. Both EndoCAb and sCD14 have been reported in four studies, with conflicting results both within and between studies (Figure 2). In one cohort of Bangladeshi infants, sCD14 measured at 6 weeks of age was negatively associated with both OPV1 seroconversion and rotavirus protection but this association became positive at week 16 (for OPV1 only) [56]. In a separate cohort of older Bangladeshi children from the same urban slum region (Mirpur), sCD14 measured on the day of vaccine receipt was positively associated with *Vibrio cholerae* LPS, implying that microbial translocation may augment immune responses to the oral vaccine. However, in the same study, EndoCAb was negatively associated with cholera toxin-specific effector memory T cells suggesting that in the context of high microbial translocation, the T-cell response to oral cholera vaccine is attenuated [57]. In southern India, neither EndoCAb nor sCD14 were associated with OPV3 seroconversion or shedding [52]. The same was true in a Zambian study, where no association was seen for either EndoCAb or sCD14 and seroconversion to RVV [55].

Tryptophan is an aromatic amino acid, which has important anti-inflammatory effects via its catabolism to kynurenine. The enzyme indoleamine 2,3-dioxygenase 1 (IDO1) mediates this pathway and is expressed on both immune and epithelial cells [66]. Indoleamine 2,3-dioxygenase 1 activity, which is reflected in the kynurenine:tryptophan (KT) ratio, therefore modulates systemic and local immune responses [67] and has been used as a marker in systemic inflammatory conditions including inflammatory bowel disease and HIV [68–70]. It has also recently been explored as a candidate marker for EED. In a Peruvian birth cohort, the KT ratio not only correlated with linear growth and systemic markers of inflammation but was also predictive of failed response to OPV1 [53]. C-reactive protein is an acute phase protein synthesized by the liver in response to the proinflammatory cytokine IL-6. However, among Bangladeshi infants in the PROVIDE study, there was no clear association between a range of proinflammatory markers (IL1-β, IL-6 and C-reactive protein) and responses to OPV or RVV [56]; IL-10, which downregulates Th1 cytokines, when grouped into quartiles was negatively associated with rotavirus IgA seroconversion if in the 50th–75th percentile but positively correlated if in the 75th–100th percentile. Interestingly, a number of the systemic biomarkers were also positively associated with responses to the parenteral tetanus vaccine.

SIBO has been implicated in the development of EED [36]. Markers of EED such as fecal Reg1β and fecal calprotectin are elevated in SIBO-positive children [36]. The gold standard diagnosis of SIBO requires sampling and culture of fluid from the intestinal lumen, which is impractical in infants. Instead, testing the hydrogen content of exhaled air is preferred as a less invasive measure of SIBO, although low sensitivity and specificity have been reported [71]. Only one study has measured SIBO in the context of oral vaccine responses. This study found an association between SIBO (measured using hydrogen breath testing) and decreased immunogenicity of the oral cholera vaccine in a cohort of Chilean children [54], even though the prevalence of SIBO in the cohort (5.6%) was lower than anticipated (20%) and lower in prevalence than vaccine failures in these populations.

It is likely that the four domains of EED interact, as depicted in Figure 1. The tight grouping of EED biomarkers (at least one from each domain) in the cluster analysis described in the PROVIDE study supports this [72]. In the Nicaragua study, fecal biomarkers of intestinal inflammation (neopterin, MPO and calprotectin) were correlated with a fecal marker of permeability (AAT) [51]. In the Indian azithromycin trial, all fecal biomarkers (neopterin, MPO, calprotectin and AAT) correlated with each other; however, they did not correlate with plasma biomarkers of intestinal epithelial damage (I-FABP) or microbial translocation (EndoCAb, sCD14) [52].

### Evidence from intervention studies

Several studies have explored interventions to augment oral vaccine performance including micronutrients, antimicrobials, probiotics and dosing strategies [73]; however, we identified only one intervention trial that measured EED biomarkers. Some EED biomarkers (calprotectin, MPO and AAT) were reduced in children randomized to a 3-day course of 10 mg/kg azithromycin compared with placebo [52]; however, this difference did not translate into improvements in OPV3 seroconversion. Other studies have examined interventions targeting EED directly, such as nutrient supplementation [74–77], anti-inflammatory agents [78], rifaximin [79] and probiotics [80]. None of these studies included a measure of vaccine response as a secondary outcome.

### Evidence from animal studies

Animal models have been used to explore the impact of several exposures on oral vaccine responses. Probiotic colonization, for example, protects against diarrhea after rotavirus challenge in gnotobiotic piglets [81]; and protein energy malnutrition has been shown to alter IgA responses to rotavirus vaccination in mice [82]. We found one animal study that explored the impact of EED on oral vaccine responses. In a gnotobiotic pig model, following three doses of oral RVV, more rotavirus-specific immune cells were detectable in pigs colonized with a healthy compared with unhealthy human gut microbiota [83]. The difference between ‘healthy’ and ‘unhealthy’ was based on two Nicaraguan infant donors with divergent EED scores and RotaTeq responses [51]; however, they failed to recapitulate the histological changes of enteropathy. A more robust model of EED was recently developed in mice, by introducing consumption of a low protein/fat diet alongside iterative oral exposure to commensal *Bacteroidales* species and *Escherichia coli* [31]. It would be valuable to further explore vaccine responses in this murine model of EED.

### Evidence from other gastrointestinal conditions

EED has some phenotypic overlap with other chronic inflammatory conditions affecting the intestine, and we therefore reviewed studies that had evaluated oral vaccine performance in the context of chronic gastrointestinal disorders.

#### Celiac disease

The changes of EED are morphologically similar to those of celiac disease, an enteropathy resulting from exposure to the food protein gluten in genetically susceptible hosts. Antibody responses to parenteral vaccines are generally unaffected [84–87] as might be expected, since these vaccines bypass the gut. One study from the 1970's describes reduced neutralizing antibody titers to OPV in patients with celiac disease [88] which was attributed to IgA deficiency; however, a later study found no impairment in the magnitude of serum IgA or IgG responses to a monovalent OPV [89]. The relevance of these results for EED is unclear, given that abnormalities of the small intestinal mucosa resolve over time in celiac disease after introduction of gluten-free diets.

#### Inflammatory bowel disease

The inflammation of Crohn's disease is transmural and variably distributed along the entire length of the intestine, rather than the confluent small bowel distribution characteristic of EED. In a 1979 study, patients with Crohn's disease given a trial treatment of oral BCG demonstrated evidence of anergy [90]. However, we found no other studies examining the response to oral vaccination in IBD. Altered immune responses to parenteral vaccines have been described in IBD patients but the immune defects are attributed to immunosuppressive treatment rather than intestinal disease [91].

#### Intestinal resection

A dramatically shortened bowel following intestinal resection reduces the mucosal surface area and may affect uptake of oral vaccines. However, RotaTeq, given to infants with a history of bowel resection, was immunogenic despite varying lengths of residual bowel [92]. A study of adults with intestinal resection reported unaltered immune responses to oral cholera vaccine when compared with age-matched healthy volunteers [93], although all had undergone colectomies due to ulcerative colitis, and the impact of small intestinal resection was not evaluated.

### Evidence from conditions overlapping with EED

Other perturbations in the intestinal milieu, overlapping and interacting with EED, may also contribute to the pathogenesis of impaired oral vaccine responses (Figure 1). First, diarrhea is common among infants in low-income countries and has been linked to reduced vaccine efficacy; OPV has consistently been shown to be less effective in children with concurrent diarrhea [94,95]. Diarrhea leads to shorter intestinal transit times, which may limit vaccine exposure. Increased mucosal innate immune responses may also impair vaccine replication and permeation. Second, enteropathogens may inhibit oral vaccines. Findings from a systematic review showed that concurrent enterovirus infection attenuates immune responses to OPV [96]. More recently, a study among Bangladeshi infants has shown that enterovirus infection at the time of vaccination impairs immune responses to RVV as well as OPV [97]. Finally, the microbiota has an important role in the development and maturation of the mucosal and systemic immune systems. A study in Ghana found differences in the composition of the bacterial microbiota between responders and nonresponders to oral RVV [98]. In prevaccination fecal specimens, increases in the Firmicutes phylum (particularly *Streptococcus bovis*) correlated with RVV seroconversion while an increased abundance of the Bacteroidetes phylum was associated with non-response. However, a recent study in Indian infants found no significant association between the bacterial microbiota composition and Rotarix seroconversion [99]. None of these studies measured EED and therefore it is difficult to determine whether vaccine response is shaped by these entities alone or through EED. However, data are beginning to emerge from larger cohorts such as the MAL-ED study [100], which may help to delineate these relationships.

## Discussion

It is biologically plausible that EED contributes to oral vaccine failure. Broadly, it also seems that population trends in EED prevalence are inversely correlated with vaccine response. However, in our review of the literature, few consistent findings emerged from existing studies, which were highly heterogeneous. Many biomarkers did not correlate significantly with oral vaccine responses; where significant associations did exist, there were often conflicting findings both between and even within studies. It therefore remains uncertain whether EED contributes to oral vaccine failure based on current data, and further studies are required.

There are likely to be a number of reasons for the heterogeneous findings. First, there were problems with study design. Two studies [51,57] included fewer than 50 subjects and were likely underpowered to detect significant differences, although power calculations were not provided. Several studies measured multiple biomarkers, sometimes at several time points, and used varying analysis methods; these high dimensional data are prone to type 1 error. Second, exploring associations with oral vaccine performance is hampered by lack of a case definition of EED. Each study selected a different group of biomarkers to define EED. Two studies [51,52] reported EED using a score based on a combination of three fecal inflammatory markers, described elsewhere [63]. Grassly *et al*. coded EED as a binary variable, which was categorized as present if one or more biomarkers measurements were in the top quartile [52]; Becker-Dreps *et al*. added a further inflammatory biomarker to the score to make a ‘4 Biomarker EED score’ [51]. Other studies used cluster analyses to highlight the most informative biomarkers [72]. Third, cross-comparison is further complicated by multiple differences between studies. For example, the characteristics of each oral vaccine differ, whether killed (oral cholera vaccines) or live (oral polio, rotavirus and typhoid vaccines) with varying strains, adjuvant properties and sites of replication (if live) in the intestinal tract. It is possible that each vaccine is influenced differently by the intestinal milieu. There are also differences in age across studies, ranging from 2 months to school age (Table 2). We believe that EED evolves over time, although we lack understanding of the exact kinetics; differences in age may be associated with substantial changes in gut architecture and function, capturing different windows in the progression of EED. Fourth, markers used to measure oral vaccine immunogenicity do not always correlate with protection from disease, which is the gold standard measure of efficacy. Rotavirus IgA seroconversion, for example, used as an outcome measure in four of the studies above, is likely a poor correlate of protection from rotavirus diarrhea in low-income countries [101]. However, measuring oral vaccine immunogenicity is often preferred to protective efficacy, which necessitates large numbers of study participants and detection of disease cases. Only one study, by Naylor *et al*., used Rotarix protection as an outcome by recording cases of rotavirus diarrhea in vaccinated infants up to 1 year of age [56]. Finally, all the studies were carried out within relatively homogeneous populations, often with ubiquitous EED; to date, an appropriate control group has therefore been lacking.

It is possible that the conflicting evidence arising from this small group of studies is because no biological association exists between oral vaccine underperformance and EED, and instead there are other explanations for why oral vaccines are less efficacious in low-income countries. It is also likely that biomarkers of EED are of limited accuracy. Nevertheless, the overlap between EED and oral vaccine underperformance is striking and the magnitude of intestinal damage when compared with children in high-income countries is high. In a Zimbabwean cohort for example, levels of I-FABP exceeded those of European children with celiac disease [102]. As our understanding of EED evolves, and the range of biomarkers available to characterize different domains of the condition expands, further well-designed studies are needed to investigate this hypothesis further.

## Conclusion & future perspective

Current evidence is insufficient to determine whether EED contributes to oral vaccine underperformance. Several factors complicating existing studies need to be resolved to clarify this important question. First, more studies are needed to inform a case definition of EED. A revised case definition has recently been proposed for use in therapeutic trials [48], which includes criteria akin to the Jones criteria for rheumatic fever, but it does not characterize all domains of the complex pathogenic pathway underlying EED [103]. The EED Biomarkers Initiative Consortium supported by the Bill & Melinda Gates Foundation is evaluating several candidate biomarkers, and in some sites work is under way to correlate biomarkers with gut biopsies or confocal laser endomicroscopic findings. Newer biomarkers such as KT ratio and citrulline should be further investigated in different cohorts especially in the context of rotavirus vaccination; work in this field is currently underway in a cohort of Zimbabwean infants [58]. Second, studies should align their selection of biomarkers, measurement time points and outcomes, to facilitate cross-site comparisons. Methods that allow for high dimensional and correlated biomarker data need to be employed [104]. Finally, it is worth considering that different oral vaccines may be impacted by different factors. Conclusions drawn from OPV failure may not apply to RVV and vice versa. The most pressing of these at present is RVV underperformance and this should be the key focus of studies going forward, including efforts to find a better correlate of protection for RVV in low-income countries.

**Box 1.**  **Outstanding research questions.**

- Does environmental enteric dysfunction (EED) reduce oral vaccine performance?
- Can prevention or treatment of EED improve responses to oral vaccines?
- Are some oral vaccines more susceptible than others to EED?
- What is the interplay between EED, diarrhea, enteropathogen carriage, composition of the microbiota and underperformance of oral vaccines?
- Would an EED point of care test identify children at higher risk of oral vaccine failure?

**Executive summary**

**Oral vaccines have reduced immunogenicity and efficacy in low-income countries**

- Oral vaccines are a vital tool for tackling enteric infections.
- However, oral rotavirus, polio, cholera and typhoid vaccines have consistently been shown to be less immunogenic and efficacious in low-income countries.
- A number of factors have been implicated but the precise reasons for underperformance remain unclear.

**Environmental enteric dysfunction**

- Environmental enteric dysfunction (EED) is a subclinical condition almost ubiquitous among children living in poverty, where oral vaccines underperform.
- It is characterized by villous blunting and mucosal inflammation as well as functional disturbances, which include increased intestinal permeability and absorptive capacity.
- The precise etiology remains unclear although microbial exposure appears to play a role.
- The definition and measurement of EED relies on noninvasive biomarkers, which capture the ‘domains’ of EED: intestinal permeability, intestinal epithelial damage, intestinal inflammation and microbial translocation.

**EED & oral vaccine failure**

- We identified eight studies describing oral vaccine responses in the context of EED across five countries.
- Four studies reported a negative association between EED and oral vaccine responses; two studies showed no significant association; and two studies concluded that EED was positively correlated with immunogenicity to oral vaccine antigens.
- Across the four domains of EED few consistent trends emerged.
- Treatment of enteropathy has not resulted in improvements in oral vaccine immunogenicity to date.
- There is no clear evidence of altered oral vaccine responses in other gastrointestinal conditions.

**Conclusion**

- Existing studies exploring the relationship between EED and oral vaccine performance are few and highly heterogeneous.
- Although it is plausible that EED contributes to oral vaccine failure, the current evidence remains insufficient.
- Further well-designed studies are needed.
