# Supplementary material for: Exploring the relationship between environmental enteric dysfunction and oral vaccine responses
Source: Future Microbiol. 2018 Jun 21;13(9):1055–70. doi: 10.2217/fmb-2018-0016 (PMC6136084; doi:10.2217/fmb-2018-0016)
Supplement: Supplementary file 1 [file fmb-13-1055-s1.docx]

**Supplementary tables (S1-4)**

**S1:** Detailed summary of evidence for association between Domain 1 and oral vaccine responses

| **INTESTINAL PERMEABILITY** | | | | | | | |
| --- | --- | --- | --- | --- | --- | --- | --- |
| **Author** | **Vaccine** | **Measure of vaccine response** | **Statistical test** | **Mannitol recovery**  Outcome details  *(P-value)* | **AAT levels**  (mg/g) | **AAT**  Outcome details  *(P-value)* | **Zonulin**  Outcome details  *(Effect size)* |
| Becker-Dreps | RV5 | Seroconversion | Wilcoxon rank sum |  | Median SC 129.5 vs non-SC 152.3ug/mL | *(P=0.4*) |  |
| Grassly | mOPV-3 | Seroconversion OPV3 | Wilcoxon rank sum |  | Median SC: 1.03 vs non-SC: 1.28 | *(P=0.126)* |  |
|  |  | Poliovirus shedding |  |  |  | *(P=0.044)* |  |
| Mwape | RV1 | Seroconversion | Poisson regression |  |  |  | Doubling zonulin  *aRR 1.24*  *(CI 1.12-1.37)* |
| Naylor | RV1 | Efficacy | Univariate linear or logistic regression followed by multivariable regression analysis with SCAD regularisation |  | Mean +/- SE  0.86 +/- 0.7mg/g |  |  |
|  | RV1 | Seroconversion |  |  |  | Measured at  12 weeks of age |  |
|  | tOPV | Seroconversion OPV1 |  | Measured at  12 weeks of age |  |  |  |
|  |  | Seroconversion OPV2 |  |  |  |  |  |
|  |  | Seroconversion OPV3 |  |  |  |  |  |
| Uddin | *Dukoral* (WC-rBS) | Vibriocidal Ab & LPS IgG, IgA, CTB IgG, IgA | MV linear regression analysis with LASSO |  |  |  |  |
|  |  | CT-specific Tem, FH, Tem B7, Tem CCR9, IFN-y, IL13, IL17, IL10 |  |  |  | CT specific IL10 only |  |

**S2:** Detailed summary of evidence for association between Domain 2 and oral vaccine responses

| **INTESTINAL EPITHELIAL DAMAGE & REPAIR** | | | | | | |
| --- | --- | --- | --- | --- | --- | --- |
| **Author** | **Vaccine** | **Measure of vaccine response** | **Statistical test** | **IFAB-P**  Outcome details  *(Effect size/P-value)* | **Citrulline**  Outcome details  *(P-value)* | **REG1B**  Outcome details  *(P-value)* |
| Grassly | mOPV-3 | Seroconversion OPV3 | Wilcoxon rank sum | *(P=0.138)* |  |  |
|  |  | Poliovirus shedding |  | *(P=0.091)* |  |  |
| Kosek | tOPV | Seroconversion OPV1 | Logistic regression adj. for LAZ, BF, ABx use & diarrhoea |  |  |  |
|  |  | Seroconversion OPV2 |  |  |  |  |
|  |  | Seroconversion OPV3 |  |  |  |  |
| Mwape | RV | Seroconversion | Poisson regression | Doubling IFABP  *aRR = 1.07*  *(CI 1.02-1.13)* |  |  |
| Naylor | RV1 | Efficacy | Univariate linear or logistic regression followed by MV regression analysis with SCAD regularization |  |  | Measured at  6 & 12 weeks of age |
|  | RV1 | Seroconversion |  |  |  |  |
|  | tOPV | Seroconversion OPV1 |  |  |  | Measured at  12 weeks of age |
|  |  | Seroconversion OPV2 |  |  |  | Measured at  6 weeks of age |
|  |  | Seroconversion OPV3 |  |  |  |  |
| Uddin | *Dukoral* (WC-rBS) | Vibriocidal Ab & LPS IgG, IgA, CTB IgG, IgA | MV linear regression analysis with LASSO | LPS IgG  *(P=0.01)* |  |  |
|  |  | CT-specific Tem, FH, Tem B7, Tem CCR9, IFN-y, IL13, IL17, IL10 |  | CT-specific TEM  *(P=0.01)* |  |  |

**S3:** Detailed summary of evidence for association between Domain 3 and oral vaccine responses

| **INTESTINAL INFLAMMATION** | | | | | | | | |
| --- | --- | --- | --- | --- | --- | --- | --- | --- |
| **Author** | **Vaccine** | **Measure of vaccine response** | **Statistical test** | **CAL levels** (ug/g) | **CAL**  Outcome details  *(P-value)* | **MPO levels**  (ug/ml) | **MPO**  Outcome details  *(P-value)* | **NEO**  Outcome details  *(P-value)* |
| Becker-Dreps | RV5 | Seroconversion | Wilcoxon rank sum | Median SC 156.2 vs non-SC 199.1 | *(P=0.03)* | Median SC: 3.1 vs non-SC: 1.1 | (*P=0.002)* | *(P=0.7)* |
| Bucardo | RV1 / RV5 | Seroconversion | Wilcoxon signed rank | 25th-75^th^ centile 2.3-9.7 | *(P=0.53)* |  |  |  |
| Grassly | mOPV-3 | Seroconversion | Wilcoxon rank sum | Mean SC 918 vs non-SC 1043 | *(P=0.191)* | Mean SC 17.4 vs  Non-SC 19.7 | *(P=0.297)* | *(P=0.757)* |
|  |  | Poliovirus shedding |  |  | *(P=0.021)* |  | *(P=0.302)* | *(P=0.779)* |
| Naylor | RV1 | Efficacy | Univariate linear or logistic regression followed by MV regression analysis with SCAD regularization | 82.7% infants with levels >200  Mean +/- SE  794.15 +/- 741.7 |  | 88.1% infants with levels >2  Mean +/- SE  10.98 +/-11.8 |  | Measured at  12 weeks of age |
|  | RV1 | Seroconversion |  |  |  |  |  |  |
|  | tOPV | Seroconversion OPV1 |  |  | Measured at  12 weeks of age |  |  |  |
|  |  | Seroconversion OPV2 |  |  |  |  |  |  |
|  |  | Seroconversion OPV3 |  |  |  |  |  |  |
| Uddin | *Dukoral* (WC-rBS) | Vibriocidal Ab & LPS IgG, IgA, CTB IgG, IgA | MV linear regression analysis with LASSO |  |  |  | LPS IgA & CTB IgG  *(P=0.02 & 0.03)* |  |
|  |  | CT-specific Tem, FH, Tem B7, Tem CCR9, IFN-y, IL13, IL17, IL10 |  |  |  |  |  |  |

**S4:** Detailed summary of evidence for association between Domain 4 and oral vaccine responses

| **MICROBIAL TRANSLOCATION & IMMUNE ACTIVATION** | | | | | | | | |
| --- | --- | --- | --- | --- | --- | --- | --- | --- |
| **Author** | **Vaccine** | **Measure of vaccine response** | **Statistical test** | **EndoCAB**  Outcome details  *(P-value)* | **Soluble CD14**  Outcome details  *(P-value)* | | **KT ratio**  Effect size  *(Confidence interval)* | **CRP**  Outcome details  *(P-value)* |
| Grassly | mOPV-3 | Seroconversion | Wilcoxon rank sum | *(P=0.074)* | *(P=0.291)* | |  |  |
|  |  | Poliovirus shedding |  | *(P=0.098)* | *(P=0.735)* | |  |  |
| Kosek | tOPV | Seroconversion OPV1 | Logistic regression adj. for LAZ, BF, ABx use & diarrhoea |  |  | | OR 1.89; 95% (*CI = 1.21–2.97)* |  |
|  |  | Seroconversion OPV2 |  |  |  | |  |  |
|  |  | Seroconversion OPV3 |  |  |  | |  |  |
| Mwape | RV | Seroconversion | Test for trend | *(P=0.073)* | *(P=0.189)* | |  |  |
| Naylor | RV1 | Efficacy | Univariate linear or logistic regression followed by MV regression analysis with SCAD regularization |  | Measured at  6 weeks of age | |  |  |
|  | RV1 | Seroconversion |  |  |  | |  |  |
|  | tOPV | Seroconversion OPV1 |  |  | 6 wks | 18 wks |  |  |
|  |  | Seroconversion OPV2 |  |  |  | |  |  |
|  |  | Seroconversion OPV3 |  |  |  | |  |  |
| Uddin | *Dukoral* (WC-rBS) | Vibriocidal Ab & LPS IgG, IgA, CTB IgG, IgA | MV linear regression analysis with LASSO |  | LPS IgG  0.002 *(P=0.07)* | |  |  |
|  |  | CT-specific Tem, FH, Tem B7, Tem CCR9, IFN-y, IL13, IL17, IL10 |  | CT-specific Tem  *(P=0.02)* |  | |  |  |

**Key for all tables**

|  | | = significant positive association |
| --- | --- | --- |
|  | | = significant negative association |
|  | | = no significant association |
|  |  | = significant negative & positive association, when marker measured at different time points |

Effect sizes and P values detailed where available.

SC= seroconverters, non-SC = non-seroconverters, NS = not specified, RV5, pentavalent rotavirus vaccine or *Rotateq*; RV1, monovalent rotavirus vaccine or *Rotarix*; mOPV3, monovalent oral polio vaccine (strain 3); tOPV, tetravalent oral polio vaccine (strain 1, 2 and 3); LPS, *Vibrio cholera* lipopolysaccharide; CTB, cholera toxin B subunit; Tem, effector memory T cells; L:M, lactulose:mannitol ratio; AAT, α1-anti-trypsin; IFAB-P, Intestinal fatty acid binding protein; CAL, calprotectin; MPO, myeloperoxidase; NEO, neopterin; EnCAB, endotoxin core antibody; sCD14, soluble CD14; KT ratio, kynurenine-tryptophan ratio; CRP, C-reactive protein; LAZ, length for age; BF, breastfeeding; ABx, antibiotic; LASSO, least absolute shrinkage and selection operator; SCAD, smoothly clipped absolute deviation; MV, multivariable

**S5: Search strategy for EED and oral vaccines**

We conducted a systematic search using *Medline* (see appendix 1) for articles published on or before 2^nd^ May 2017 describing vaccine responses in the context of EED (search A-D). Abstracts and titles from all years were compiled in Endnote (Thomson Reuters) and screened. The search was subsequently broadened also to include oral vaccine responses in the context of other gastrointestinal conditions with phenotypic features in common with EED (search E-F). Our review was restricted to articles either written in English or translated into English. Unpublished data were not included.

1. Intestinal inflammation
2. Intestinal permeability and MT
3. Villous atrophy and absorptive capacity
4. SIBO or SBBO or small bowel overgrowth

**Search 1 – Search all**

(vaccine OR immunisation OR immunization) AND (environmental enteropathy OR environmental enteric dysfunction OR tropical sprue OR tropical enteropathy) – 2^nd^ May 2017 (HIT: 445)

**Search A**

((oral OR enteric) AND (vaccine OR immunisation OR immunization)) OR rotavirus vaccine OR polio vaccine OR cholera vaccine AND ((intestin* or gut or mucosa*) AND inflammation)) – 2^nd^ May 2017 (HIT: 338)

**Search B**

((oral OR enteric) AND (vaccine OR immunisation OR immunization)) OR rotavirus vaccine OR polio vaccine OR cholera vaccine AND ((intestin* OR gut OR mucosa*) AND (permeability OR microbial translocation)) – 2^nd^ May 2017 (HIT: 104)

**Search C**

((oral OR enteric) AND (vaccine OR immunisation OR immunization)) OR rotavirus vaccine OR polio vaccine OR cholera vaccine AND ((Villus AND (atrophy or blunting or flattening)) OR absorptive capacity OR surface area) – 2^nd^ May 2017 (HIT: 157)

**Search D**

vaccine OR immunisation OR immunization AND ((small bowel overgrowth) OR (bacterial overgrowth syndrome) OR SIBO OR SBBO)) – 2^nd^ May 2017 (HIT: 6)

**Search E**

vaccine OR immunisation OR immunization AND (coeliac or celiac) – 2^nd^ May 2017 (HIT: 202)

**Search F**

((oral OR enteric) AND (vaccine OR immunisation OR immunization)) OR rotavirus vaccine OR polio vaccine OR cholera vaccine AND (inflammatory bowel disease OR crohns OR colitis OR ulcerative colitis) – 2^nd^ May 2017 (HIT: 202)
